# All-dielectric ultrathin conformal metasurfaces: lensing and cloaking applications at 532 nm wavelength

**DOI:** 10.1038/srep38440

**Published:** 2016-12-08

**Authors:** Jierong Cheng, Samad Jafar-Zanjani, Hossein Mosallaei

**Affiliations:** 1 Department of Electrical and Computer Engineering, Northeastern University, 360 Huntington Avenue, Boston, Massachusetts 02115, USA

## Abstract

Metasurfaces are ideal candidates for conformal wave manipulation on curved objects due to their low profiles and rich functionalities. Here we design and analyze conformal metasurfaces for practical optical applications at 532 nm visible band for the first time. The inclusions are silicon disk nanoantennas embedded in a flexible supporting layer of polydimethylsiloxane (PDMS). They behave as local phase controllers in subwavelength dimensions for successful modification of electromagnetic responses point by point, with merits of high efficiency, at visible regime, ultrathin films, good tolerance to the incidence angle and the grid stretching due to the curvy substrate. An efficient modeling technique based on field equivalence principle is systematically proposed for characterizing metasurfaces with huge arrays of nanoantennas oriented in a conformal manner. Utilizing the robust nanoantenna inclusions and benefiting from the powerful analyzing tool, we successfully demonstrate the superior performances of the conformal metasurfaces in two specific areas, with one for lensing and compensation of spherical aberration, and the other carpet cloak, both at 532 nm visible spectrum.

Metasurfaces are optically thin layers of two-dimensional (2D) arrangement of subwavelength scatterers for local manipulation of electromagnetic waves^1^,^2^,^3^. They have enabled a variety of superior functionalities in a planar and compact manner^4^,^5^,^6^,^7^,^8^,^9^,^10^. Benefiting from the advances of nano-manufacturing techniques, metasurfaces are becoming competitive candidates for replacing conventional bulky optical devices. On the other hand, there is a strong demand for engineering the performance of existing devices without changing their physical geometries. The functionality and geometry of several conventional devices (lenses, mirrors, waveplates, etc) are correlated in a way that desired phase profile is accumulated along the optical path. By coating a metasurface layer, the optical performance can be decoupled from the geometry and further engineered at the surface, leading to richer functionalities, relaxed fabrication requirements, and increased freedom for other considerations such as mechanical and aerodynamic features^11^.

The 2D nature of the metasurfaces makes them naturally suitable to be transferred onto and wrapping around the existing systems, especially when the surfaces have curved appearance. Several studies have been performed on conformal metasurfaces coating the microwave antennas for improvement of the radiation patterns^12^,^13^. Flexible metasurfaces with plasmonic inclusions for invisibility cloaking from terahertz to infrared and even visible regime have been reported recently^14^,^15^,^16^. Here we study the conformal metasurfaces made of dielectric disks embedded in a flexible thin layer at visible frequency range (532 nm) for different optical applications. The inclusions can provide desired phase shift from 0 to 360° with high efficiency by properly tuning the fundamental electric and magnetic resonances. The all-dielectric design offers better efficiency and stronger power tolerance due to the absence of Ohmic loss especially at visible frequencies compared to the plasmonic counterparts. The disk inclusions are less sensitive to the incidence angle due to their small thickness, compared to the dielectric nanopost waveguides recently studied in refs 11, 17 where the thickness is comparable to the operating wavelength. This feature makes the proposed metasurface also robust enough to conform to various platforms without significant performance degradation.

An efficient modelling technique hybridizing the finite-difference time-domain (FDTD) method and Green’s function based on the field equivalence principle^18^,^19^ is proposed for solving large arrays of metasurface nanoantennas on conformal platforms, which are prohibitively challenging for the conventional full-wave numerical techniques. While the field equivalence principle is well illustrated in many electromagnetic textbooks, it has not been discussed in the characterization of metasurfaces at the high frequency regime and with conformal shapes where the effective surface currents are not readily available. The hybridization of FDTD and Green’s function is systematically developed by taking care of the subwavelength couplings and interactions while keeping high efficiency in dealing with large-scale metasurface problems. The aforementioned method is then utilized to efficiently design two types of metasurfaces working in transmission and reflection modes at *λ* = 532 nm, with the thickness of only *λ*/6 and *λ*/13, respectively. Promising performance is observed and discussed in terms of correcting spherical aberration in commercial lenses and realizing optical carpet cloaking.

## Results

**Figure 1:**
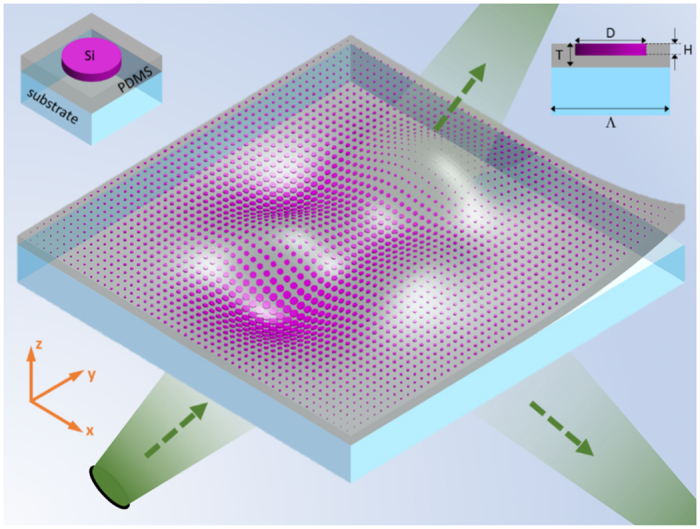
Schematic of a conformal metasurface wrapping onto an arbitrarily curved platform. The 3D and the cross-section views of the unit cell are shown in the upper left and right corners, respectively, along with materials and parameters defined in this study.

A conformal metasurface wrapping onto an arbitrarily curved platform is schematically shown in Fig. 1. Typically, the platform is extremely large (millimeter scale) with a curved surface covered by a huge subwavelength nanoantenna array for electromagnetic transformation, making the design and modelling challenging. The conformal metasurface array under such circumstances can be approximated by locally flat inclusions with slow space-variation. Furthermore, given the high working frequency, the incident fields can be locally considered as a plane wave^19^ with space-variant incidence angles. With the aforementioned assumptions, the performance of the metasurface can be considered as a collective behaviour with each inclusion functioning as if it is in a flat periodic array. The local response of each nanoantenna can be readily characterized through numerical simulations by considering a flat and periodic unit cell with proper plane wave excitation. A hybrid FDTD/Green’s function method based on the field equivalence principle is used to design the local phase controllers, gather the equivalent surface currents over the metasurface array, and tranform into the far field distributions.

### Conformal Metasurface Design Principles

Parametric study of the metasurface elements in a 2D periodic environment with plane wave excitation is performed using FDTD method as functions of disk diameters, polarizations and incident angles, so that one can define an optimum geometry for each element covering the curvy platform in order to locally transform the incoming wave in a desired shape.

Both plasmonic and dielectric resonators are qualified as metasurface elements for wavefront construction. The focus of this work is dielectric ones as they show a highly efficient performance at the visible regime by spectrally overlapping the electric and magnetic dipole resonances^20^,^21^,^22^. More importantly, they have additional merits of low sensitivity to the period variation and good tolerance of the incidence angle, which are necessary features as the metasurface adheres to the curved platform. The resonant performance does not change with small variations of the period, since the field is mainly confined inside the disk. The robust response to the incidence angle comes from the thin thickness of the disks, which effectively avoids resonances in the height direction. In this regard, dielectric disks behave much better than dielectric posts, due to the significant reduction of the thickness. In the latter design the phase shift is achieved through a waveguide mode in a wavelength-thick post^11^, which restricts the thickness from the physical mechanism.

In this study, the high-contrast dielectric disk resonators made of silicon (Si) embedded in a flexible polydimethylsiloxane (PDMS) layer are chosen as the conformal metasurface inclusions, whose 3D and cross-section views are shown in the upper left and right corners of Fig. 1. In terms of the possible realization process, Si disks can be fabricated on a flat substrate through lithography and etching, followed by the spin-coating of the PDMS layer. Then, the PDMS layer with the disks embedded inside can be peeled off and transferred onto the desired platform^23^,^24^. The low modulus and surface energy of PDMS allow conformal contact with the platform surface through Van der Waals interactions^25^.

**Figure 2:**
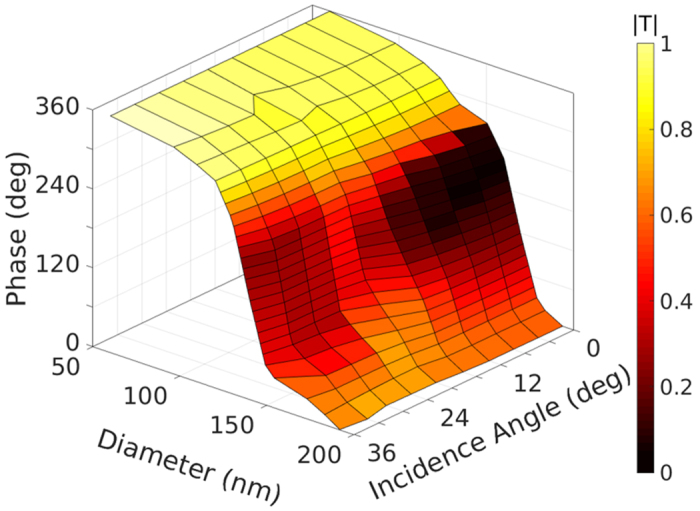
Transmission responses of dielectric disk elements with variation of diameters and oblique incidence angles in *s* mode. Period and thickness are fixed as Λ = 280 nm and *H* = 90 nm, respectively.

The substrate under the PDMS layer is considered as glass to work in the transmission mode. Targeting the visible wavelength of 532 nm, the period Λ is fixed as 280 nm, and the thickness of the disk *H* is 90 nm (*λ*/6). The permittivities of 2.0 and 2.3 are used for PDMS and N-BK7 glass, respectively, around 532 nm with low dispersion, and a dispersive permittivity model following^26^ is considered for Si. Due to the good impedance matching between PDMS and glass, the thickness of the PDMS layer *T* does not affect the element performance, and can be determined totally by experiment. Considering the azimuth symmetry of the disk inclusions, the performance is independent of the associated incidence plane. The excitation can be decomposed into *s* and *p* polarization modes with electric field normal and parallel to the incidence plane. Tuning the disk diameter *D* leads to different transmission phases under a certain incidence angle, as summarized in Fig. 2. Any phase shift within 360° is achievable with proper disk diameter ranging from 50 nm to 200 nm in the *s* mode. Such performance can be maintained quite well when the incidence angle goes up to 36°. The transmission efficiency is shown as the pseudo-color in the 3D plot of Fig. 2. The geometry is optimized for incidence angle of 20°, where the efficiency is kept above 0.4 for all the transmission phases. As the incidence angle deviates away from 20°, the efficiency degrades in the phase range of [150°, 270°]. As will be shown in the applications section, this will lower the efficiency of the device but still provide accurate phase functionality. As the inclusions are optimized in a way to tolerate large incidence angle in the *s* polarization mode, they do not behave well in providing rich phase information in the *p* polarization mode. Instead, if both *s* and *p* polarizations are responsible for wavefront engineering, our study shows that such elements keep satisfactory performance in terms of transmission phase and efficiency for incidence angle of up to 10°.

**Figure 3:**
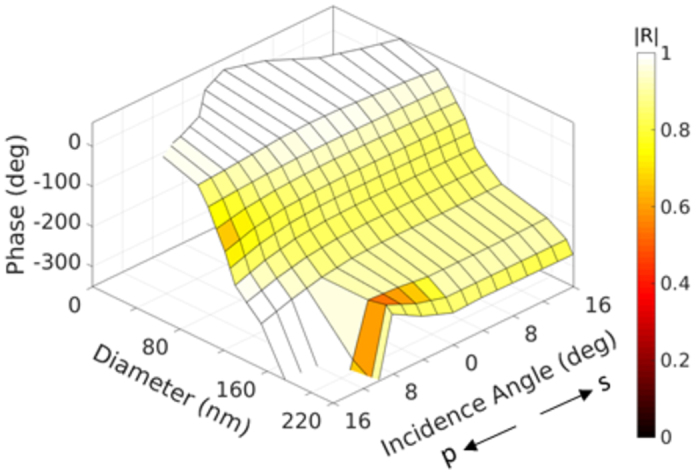
Reflection responses of dielectric disk elements grounded by a 90 nm silver layer. More than 360° of phase shift is achievable when diameters are changed in both *s* and *p* polarizations with the incidence angles up to 16°. Period and thicknesses for disk and PDMS layer are fixed as Λ = 240 nm, *H* = 30 nm, and *T* = 40 nm, respectively.

The metasurface inclusions can serve as phase controllers in the reflection mode by simply selecting the substrate as a metallic layer. Here the substrate is chosen as silver with dispersive permittivity following^27^ (*ε* = −11.76 + 0.37 at 532 nm). The thickness of the silver layer is 90 nm leading to the reflection coefficient above 0.99 at 532 nm without disks. In order to keep high efficiency when adding the disk resonator, one does not need to spectrally overlap electric and magnetic resonant modes. Instead, the highly reflective substrate and the low-loss resonators enable high reflection efficiency and large phase shift by merely engineering the fundamental mode. This further reduces the thickness of the disks compared to the ones working in the transmission mode, making the elements more robust for oblique incidence. Here the periodicity Λ is 240 nm with disk thickness *H* of 30 nm and PDMS layer thickness *T* of 40 nm (*λ*/13). The response in Fig. 3 smoothly covers more than 360° of phase shift by choosing the proper disk diameter, with reflection efficiency of more than 0.8 as indicated by the pseudo-color. Such performance remains intact for both *s* and *p* polarizations up to incidence angle of 16°. Most importantly, the plot in Fig. 3 is symmetric about the normal incidence except for the small area in the corner, which means that the designed inclusions provide same phase responses for both *s* and *p* polarizations under the same incidence angle. The corner area in *p* polarization with low efficiency and unsymmetrical phase response with respect to the one in *s* polarization does not influence the performance of the large-array metasurface application as will be illustrated in the cloaking design. For incidence angles of larger than 16°, the performance of the elements keeps well for the s polarization, but gradually deteriorates for the p polarization.

**Figure 4:**
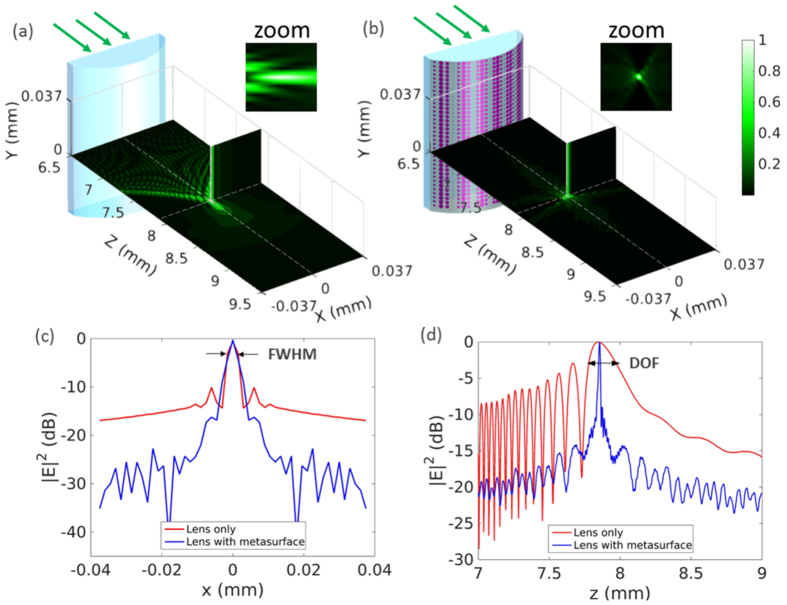
Normalized intensity distribution when light goes through the cylindrical lens without (**a**) and with (**b**) metasurface coating. The zoom-in view of each focal point in x-z plane is shown on the right corner. (**c**) Intensity comparison in *dB* along the transverse *x* direction at the focal plane. (**d**) Intensity comparison in *dB* along the optical axis.

To design the metasurface and evaluate its performance in providing a specific phase discontinuity function on a curvy substrate, we comply with the following procedures: 1) calculate the inhomogeneous phase shift profile for a specific application, and find the discretized phase shift for each meta-element, 2) analyze the incidence angle for each meta-element based on the excitation and the comformal surface shape, and decompose the excitation into *s* and *p* modes through global to local coordinate transformation, 3) find the disk diameter and the efficiency of the specific element that fulfills the phase requirement based on the numerical data in Figs 2 and 3, 4) calculate the total electric and magnetic fields on the metasurface, find equivalent surface currents and field distribution based on the field equivalence principle. The study of metasurfaces as correctors of spherical aberration in commercial cylindrical lenses and optical carpet cloak at 532 nm is carried out, while more sophisticated optical applications can be explored in a similar manner.

### Compensation of Spherical Aberration in Commercial Cylindrical Lenses

Cylindrical lenses with circular shape along one axis focus light into a line instead of a spot, which offer many application options including one-dimensional beam shaping, image stretching^28^, laser line generation for barcode scanning, collimation and circularization of diode laser outputs. For a mathematically perfect imaging, one needs a parabolic shaped lens. Spherical aberration existing due to a spherical surface describes the phenomena that the paraxial rays and marginal rays do not converge to the same point. It is a critical detriment hindering the formation of a sharp image. Spherical aberration can be minimized by either changing the spherical shape into aspherical one along the transverse axis or by cascading several lenses for aberration cancellation, which leads to high-cost production and bulky system.

Instead, spherical aberration can be compensated by coating a conformal metasurface layer on the lens with the resonator inclusions designed above. We consider a commercial plano-convex cylindrical lens (Edmund Optics #45-981) with the diameter of 5 mm and focal length of *F* = 8 mm, and transfer the metasurface layer onto its convex surface with the radius *R* = 4.13 mm. The lens is made of N-BK7 glass with the refractive index of *n*_*g*_ = 1.52 around 532 nm. The center and edge thickness are 3 mm and 2.12 mm, while the metasurface thickness is only around 100 nm.

To correct spherical aberration, the required phase shift profile within the metasurface is derived as follows based on ray optics.


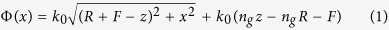


where 
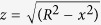
, and *k*_0_ is the wavenumber at 532 nm. The lens and the metasurface are uniform along *y* direction. *S*-polarized plane wave excitation from the planar side of the lens is propagating along *z* direction, as illustrated in Fig. 4. All the elements experience s-polarized plane wave excitation with space-variant incidence angles from 0 to 35°. By looking up the data in Fig. 2, one can find the diameter distribution, transmission amplitude and phase responses of the metasurface array.

Considering the large radius of the curved lens surface, we define a supper cell composed of 5 by 5 disk elements with exactly the same geometries, so that the local periodicity assumption holds true. The metasurface is composed of 3,570 supper cells along *x* direction and 1,420 supper cells along *y* direction with space-variant disks covering the entire lens. The hybrid FDTD/Green’s function method works efficiently and accurately in dealing with such large array and on the curved substrate. Figure 4(a) and (b) compare the focusing behaviour of the cylindrical lens without and with metasurface coating. The normalized intensity in *x*-*z* plane and *x*-*y* plane passing through the focal point are presented. Both focus into a line around 8 mm, where the *z* axis starts from the lens. The external caustic lines and the long focusing tail in Fig. 4(a) are apparent evidences of spherical aberration. In contrast, the focal point in Fig. 4(b) is much more concentrated due to the accurate phase realization in the metasurface. Further comparisons on the white dashed lines along *x* and *z* directions are depicted in Fig. 4(c) and (d). As observed from Fig. 4(c), the full width at half maximum (FWHM) of the focal spot is almost constant (3 *μ*m) for both cases, as the aperture size does not change. The metasurface lowers the sidelobe level by 6 dB and lowers the background intensity by more than 13 dB. Along the propagation direction, the depth of field (DOF) is measured in Fig. 4(d) as 195 *μ*m and 13 *μ*m for the lens only and the lens with metasurface coating. A significant improvement of the focusing quality by a factor of 15 is observed. At the focal point, the real efficiency of the metasurface layer is 58% and the relative efficiency is 214%, where the former is defined by the intensity ratio when the meta-elements have practical transmission amplitude in Fig. 2 and ideal amplitude of 1, and the latter is defined by the intensity ratio of coated and uncoated lenses. The metasurface layer minimizes spherical aberration with improved spot intensity compared to the original lens.

**Figure 5:**
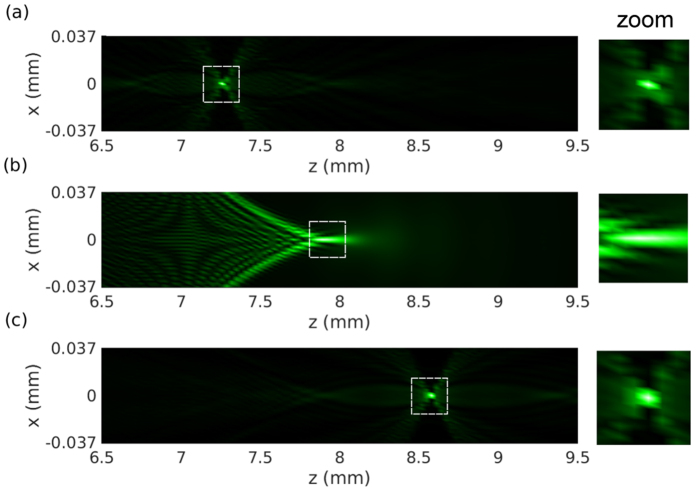
The focal length is modified into 7.2 mm in (**a**) and 8.5 mm in (**c**) with minimized spherical aberration by coating two types of metasurface layers with proper disk diameter profiles, compared to the performance of the original lens in (**b**). The field within the dashed square is zoomed in on the right for each case.

Moreover, taking advantages of the rich phase functions achievable in conformal metasurfaces, they are cost-effective choices for compensation of the fabrication error or tuning the focal length. Figure 5 shows that the focal length can be moved either smaller in Fig. 5(a) or larger in Fig. 5(c) than the original one in Fig. 5(b) with minimized spherical aberration as shown in the zoomed view. The focal length is tuned into 7.2 mm and 8.5 mm in Fig. 5(a) and (c) by coating the metasurface layer with proper diameter profile based on the element study in Fig. 2.

It is noteworthy that recently there has been also a work^11^ on phase engineering of a cylindrical lens. But as mentioned earlier authors consider nanoposts waveguides working based on a totally different concept requiring large thickness comparable to wavelength, which may not be very favorable for flexible-conformal applications, and furthermore it may create more limitation on the system functionality to oblique incidence angle operation due to the large height of the posts. Also the operating wavelength has been in infrared (at 915 nm). The presented work here is a layer of ultrathin flexible metasurface at visible band based on a totally different concept.

### Carpet Cloaking at 532 nm

The idea of using metasurfaces for carpet cloaking was proposed firstly in ref. 15, and followed by the experimental demonstration at the edge of the visible spectrum^14^. The reported metasurfaces are composed of plasmonic resonators as phase shifters to locally reconstruct the wavefront. Compared to the schemes based on transformation optics^29^ and scattering cancellation^30^, metasurfaces avoid the extreme parameter requirements and the bulky configurations. They isolate the object from the impinging wave by inhomogeneous phase discontinuity happening through a deep-subwavelength thin layer. Different from the previous cloaking works composed of plasmonic metasurface arrays and at 730 nm^14^, here we design an all-dielectric conformal cloak working at 532 nm visible band.

The cloak is designed to conceal a curved silver surface with several Gaussian bumps by regenerating the reflected wave front (both amplitude and phase) from a flat silver plane, with known plane wave excitation. The scattering surface shape is defined by scaling the absolute value of the peak function in MATLAB as *h* = 0.3|peaks(40 *μm*)|, where the dimensions along *x* and *y* directions are 40 *μ*m. The PDMS layer with embedded Si disk array is transferred onto the curved surface with the thickness of only *λ*/13, which makes it ideal to conform to objects with complex geometry and to keep low-profile. The inhomogeneous phase discontinuity profile for the metasurface follows Φ(*x, y*) = −2*k*_0_*h*cos *θ* + *π*, where the first term is the propagation phase delay compared at each specific point on the rough surface when light is reflected by the ideal flat plane and the curved one with incidence angle of *θ*, and the second term is the phase jump of the reflection at the flat silver surface. One can also derive the phase function based on geometrical optics and generalized Snell’s Law^31^.

**Figure 6:**
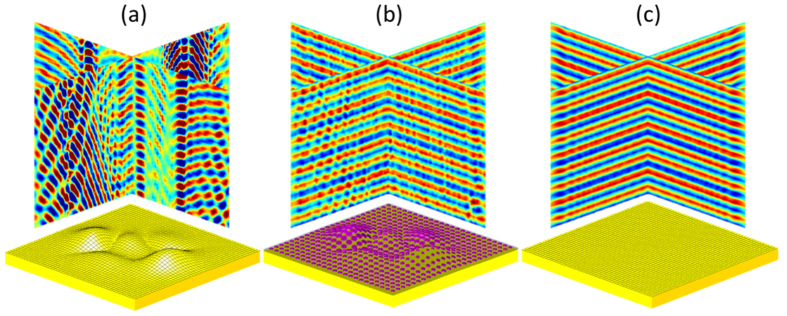
Reflected electrical field distribution above the scattering surface in (**a**) and above the same surface cloaked by the metasurface layer in (**b**). (**c**) Reflected electrical field distribution above a flat silver surface.

The cloak is composed of an array with 160 by 160 disks, impinged by a plane wave in the normal direction. The wave vector and electric field are transformed to each disk at its local coordinate system. Each disk experiences different incidence angle based on its location at the surface. Combining the required phase and the incidence angle for both *s* and *p* polarizations, one finds the disk diameter and the reflection coefficients from the FDTD data pool in Fig. 3. The reflected field distribution (real part of the electric field) above the cloak is calculated in Fig. 6(b). As a comparison, the results above the uncloaked scattering surface and the flat surface are shown in Fig. 6(a) and (c) by considering the Snell’s Law of reflection at each point on the surface. The metasurface cloak maintains the planar wave front for the reflected field with approximately the same phase and amplitude as those from a flat metallic surface, so that the receiver cannot detect any information about the concealed objects. The same color bar is used in all the plots in Fig. 6. The cloak works well with high efficiency of above 95% even though some of the cloaking elements show different phase response for *s* and *p* polarizations.

Although the proposed conformal metasurfaces work in a relatively narrow spectral region, the techniques for broadband metasurface design^32^ can be incorporated to increase the bandwidth, while the concept and the modelling methods still hold true.

In summary, conformal metasurfaces composed of all-dielectric nanoantenna inclusions with ultra-small thickness for wave engineering on curved platform at 532 nm visible spectrum are studied. The inclusions have subwavelength dimensions to enable accurate inhomogeneous phase discontinuity and associated design requirements. They in general lead to considerably large non-periodic arrays where computation becomes extremely expensive. An asymptotic modeling method is generalized to handle this issue by hybridizing the local responses from FDTD simulation and the Green’s function with promising efficiency and accuracy. A conformal metasurface with thickness of *λ*/6 is implemented to correct the spherical aberration of a commercial cylindrical lens, enabling reduction of depth of field by a factor of about 15. For the second application, an even thinner metasurface layer (*λ*/13) is designed and conformed to a rough silver surface for cloaking purposes in the visible regime. Careful regeneration of the phase of the reflected fields by the conformal metasurface results in object to be free of detection. With the proposed metasurfaces and also the developed powerful modelling scheme, manipulating electromagnetic waves in novel ways and for other sophisticated systems can be anticipated.

## Methods

Considering orders of magnitude difference between the platform size and the metasurface element dimension, the metasurface arrays for lensing and cloaking are composed of more than 126 million and 25 thousand unit cells, respectively. It is totally impossible and inefficient to design and analyze such electromagnetically huge structures with full-wave numerical methods, due to the restricted computational resources at hand. Techniques such as geometrical optics (ray-tracing) are well-established for solving similar problems in the microwave range. Here a more accurate method based on the field equivalence principle is adopted for efficient design and characterization of the problem in Fig. 1, by considering the coupling and local resonances of the inclusions up to visible spectrum.

### Field Equivalence Principle

**Figure 7:**
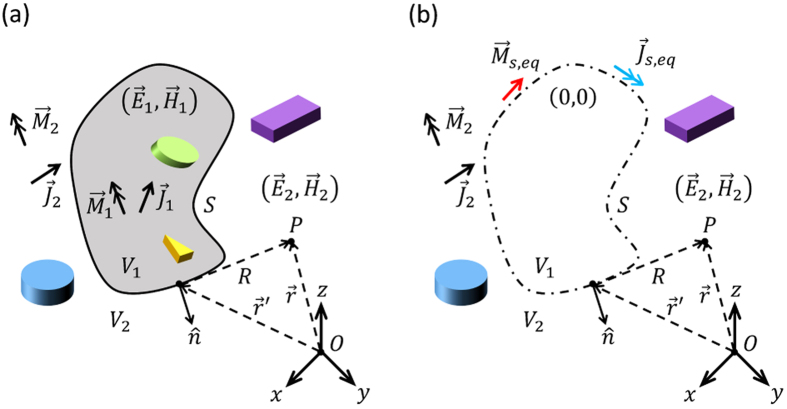
The Field Equivalence Principle. (**a**) The entire space is divided into two non-overlapping partitions. (**b**) In order to calculate the electromagnetic fields in the exterior region, geometry and sources of the interior can be replaced by a set of surface electric and magnetic currents impressed on the boundary.

Suppose that the entire space is divided into two non-overlapping partitions by a closed surface *S* (Fig. 7). Let us denote the interior region by *V*_1_, and the exterior by *V*_2_. According to the field equivalence principle (a formal representation of the Huygens-Fresnel principle^33^), if one is only interested in the electromagnetic fields in the exterior region (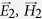), the geometry and sources in the interior can be removed and replaced by a set of surface electric and magnetic currents impressed on the boundary of the two regions^34^. More rigorously, the field equivalence principle can be expressed by an illustrative form known as the Kottler’s formulation^33^:


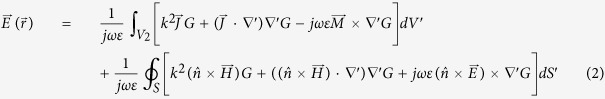



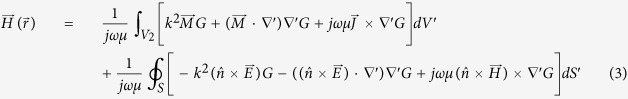


where, 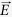 and 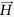 represent total electric and magnetic field intensities, *ω* is radian frequency, *ε* and *μ* are electric permittivity and magnetic permeability of the background medium, *k* is the wavenumber, and 
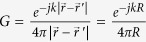
 is the scalar Green’s function of free-space. Also, primed and unprimed variables denote source and observation spaces [see Fig. 7]. Detailed justification of the above relations can be found in ref. 33. It is noteworthy that, in Eqs (2) and (3), if 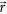 is chosen in the interior region (*V*_1_), the calculated fields will be identical to zero (extinction theorem)^33^. In other words, the aforementioned relations are only valid for obtaining the fields outside the closed surface *S*.

By comparing volume and surface integrals of Eqs (2) and (3), one can define a set of equivalent surface currents [see Fig. 7(b)]


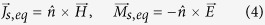


These surface current densities do not necessarily have any physical counterparts. To recap, the whole sources and geometries inside the surface can be replaced by a set of fictitious equivalent surface current densities to calculate the filed at an observation point in the exterior region.

Yet, there are some practical issues in solving real-world problems by this method. First of all, we are interested in problems involving open boundaries in lots of scenarios including Fig. 1. For instance, radiation characteristics of an aperture antenna. It is a very well-known concept in antenna engineering^33^ that, as the aperture dimension of a radiator becomes larger (typically larger than a wavelength), calculating the integrals in Eqs (2) and (3) only on the finite aperture of the antenna gives a very accurate prediction of the radiated fields, particularly, the main lobe of the radiation pattern.

Secondly, the total field intensities at the boundary S are usually the unknowns we are looking for and we do not have them in the first place to calculate the equivalent currents. However, it might be possible to calculate an acceptable local approximation of total fields given the inherent characteristic of the problem. Due to the large radii of curvature of the surface, compared to the wavelength, the conformal metasurface array can be approximated by locally flat inclusions experiencing proper plane wave excitation^19^.

### Hybrid FDTD/Green’s Function Method

Hybrid FDTD/Greens’s function, incorporating the field equivalence principle and the FDTD numerical element study, is an asymptotic method that takes advantage of the high frequency nature of the problem to solve the structures similar to the one shown in Fig. 1 efficiently, using a reasonable amount of computational resources with very good accuracy.

**Figure 8:**
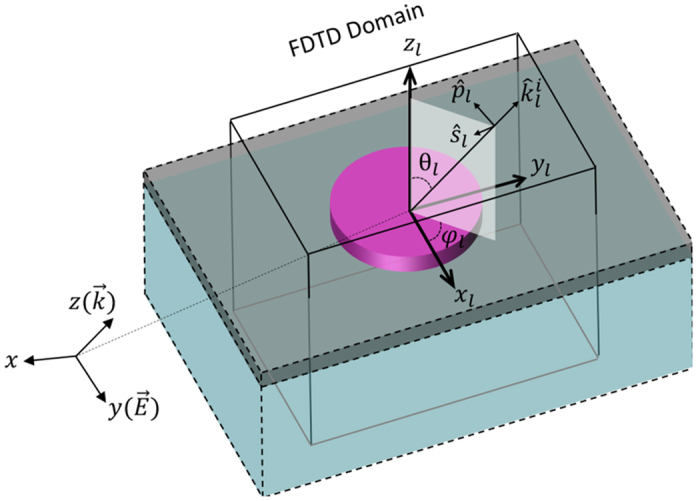
Schematic of a unit cell for coordinate transformation between the global and local coordinate systems. The incident wave is propagating along *z* with electric field along *y* in the global coordinate system. The local vector *z*_*l*_ is the normal vector to the surface at the specific element position. *x*_*l*_ and *y*_*l*_ are arbitrarily defined perpendicular to *z*_*l*_. 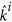 is the local incident wave-vector and 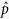, 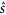 are unit vectors representing parallel and perpendicular polarizations, respectively.

Coordinate system (CS) transformation serves as bridge between the global metasurface array and the local elements (with detailed explanations in the Supplementary document). The local CS can be defined in a way that 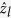 is the normal vector to the locally flat subaperture. 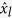, and 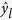 can be chosen arbitrarily in the tangential plane, as shown in Fig. 8, which does not affect the final results. The excitation information (electric field vector and wave-vector) is transformed from the global CS of the metasurface to the local CS of each nanoantenna as 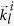 and 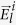 as shown in Fig. 8. At this stage, 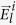 should be decomposed to *p* and *s*-polarizations with electric field parallel and perpendicular to the incident plane, which is basically a Cartesian to spherical transformation as discussed in the Supplementary document.

The incident field in the local CS can be written as ref. 19


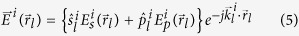



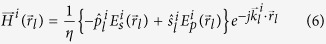


where, *η* is the wave-number of background medium. One can readily obtain the transmitted fields as


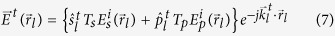



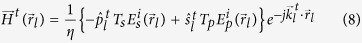


Also, reflected fields can be represented in a similar manner as


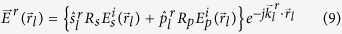



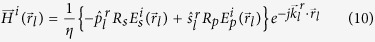


*T*_*s*_, *T*_*p*_ and *R*_*s*_, *R*_*p*_ are the transmission and reflection coefficients for the *s* and *p*-polarizations, respectively, which are characterized numerically in the designing process in terms of different incidence angles, polarizations, and geometries.

Finally, the transmitted or reflected fields should be transformed back to the global CS for the calculation of equivalent surface currents by Eq. (4). This is under the assumption that the equivalent surface currents are uniform on each nanoantenna subaperture, which can be readily characterized by a transmission or reflection coefficient. In this case, integration in the Kottler’s formula is replaced by summation over all the nanoantennas, which significantly simplifies the modelling.

## Additional Information

**How to cite this article**: Cheng, J. *et al*. All-dielectric ultrathin conformal metasurfaces: lensingand cloaking applications at 532 nm wavelength. *Sci. Rep.* **6**, 38440; doi: 10.1038/srep38440 (2016).

**Publisher's note:** Springer Nature remains neutral with regard to jurisdictional claims in published maps and institutional affiliations.

## Supplementary Material

Supplementary Information
