# Supplementary material for: All-dielectric ultrathin conformal metasurfaces: lensing and cloaking applications at 532 nm wavelength
Source: Sci Rep. 2016 Dec 8;6:38440. doi: 10.1038/srep38440 (PMC5144014; doi:10.1038/srep38440)
Supplement: Supplementary Information [file srep38440-s1.pdf]

# Supporting Information

# All-dielectric ultrathin conformal metasurfaces: lensing and cloaking applications at 532 nm wavelength

Jierong Cheng[1], Samad Jafar-Zanjani[1] & Hossein Mosallaei[1*]

[1]Department of Electrical and Computer Engineering, Northeastern University, 360 Huntington Avenue, Boston, Massachusetts 02115, USA

[*] hosseinm@ece.neu.edu

The field equivalence principle provides a convenient way to calculate the observation field with the knowledge of the total field at the metasurface boundary. Due to the slow spatial variation of the metasurface array, the total field information can be approximated with very good accuracy as the collection of the local field responses of the elements when they are uniform arrays. Communication is inevitable between the global metasurface array and the local uniform array of each element through coordinate system (CS) transformation.

Specifically, we need to transform the excitation fields ($\vec{E}$ and $\vec{H}$) and the wavevector ($\vec{k}$) from the global coordinate to the local one for the element study. To obtain the equivalent current profile of the metasurface, the local field response needs to be transformed back to global CS. Here, we try to present some key relations about such transformation. The interested reader is urged to consult [1,2] for a more thorough discussion.

Suppose that we have a vector $\vec{v} = [v_x, v_y, v_z]^t$ in the global CS $C_g\{\hat{x}, \hat{y}, \hat{z}\}$ ("$t$" denotes transpose operator). and the local CS $C_l\{\hat{x}_l, \hat{y}_l, \hat{z}_l\}$ [see Fig. 1].

$C_g$-representation of $\vec{v}$ can be mapped to its $C_l$-representation by a unitary coordinate transformation matrix (CTM) [1]:

$$\begin{bmatrix} v_{x_l} \\ v_{y_l} \\ v_{z_l} \end{bmatrix} = {}^{C_l}A^{C_g} \cdot \begin{bmatrix} v_x \\ v_y \\ v_z \end{bmatrix} \tag{1}$$

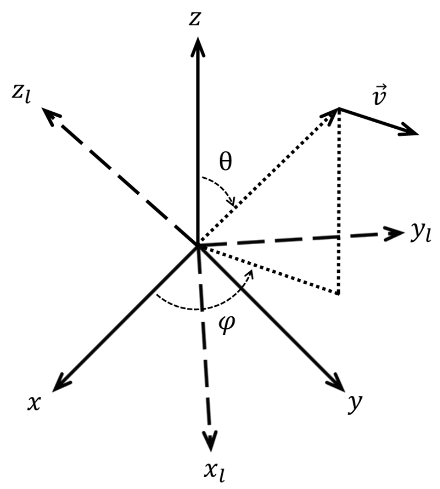


Figure 1: Coordinate system transformation.

where,

$$^{C_l}A^{C_g} = \begin{bmatrix} \hat{x}_l \cdot \hat{x} & \hat{x}_l \cdot \hat{y} & \hat{x}_l \cdot \hat{z} \\ \hat{y}_l \cdot \hat{x} & \hat{y}_l \cdot \hat{y} & \hat{y}_l \cdot \hat{z} \\ \hat{z}_l \cdot \hat{x} & \hat{z}_l \cdot \hat{y} & \hat{z}_l \cdot \hat{z} \end{bmatrix} \tag{2}$$

The transformation from local CS to global CS can be done similarly. As an example, Cartesian coordinates of a vector in a CS, $[v_x, v_y, v_z]^t$, can be transformed to its spherical coordinates, $[v_r, v_\theta, v_\phi]^t$, in the same CS using the following CTM [1,2]:

$$^{S}A^{C} = \begin{bmatrix} \sin\theta\cos\phi & \sin\theta\sin\phi & \cos\theta \\ \cos\theta\cos\phi & \cos\theta\sin\phi & -\sin\theta \\ -\sin\phi & \cos\phi & 0 \end{bmatrix} \tag{3}$$

Each CTM can be realized by applying three consecutive elemental CTMs [1]. An elemental

CTM represents the rotation of a CS, by a specific angle (e.g. $\psi$), about one of its axes:

$$ {}^{C_2}{A_x}^{C_1}(\psi) = \begin{bmatrix} 1 & 0 & 0 \\ 0 & \cos\psi & \sin\psi \\ 0 & -\sin\psi & \cos\psi \end{bmatrix} \tag{4a} $$

$$ {}^{C_2}{A_y}^{C_1}(\psi) = \begin{bmatrix} \cos\psi & 0 & -\sin\psi \\ 0 & 1 & 0 \\ \sin\psi & 0 & \cos\psi \end{bmatrix} \tag{4b} $$

$$ {}^{C_2}{A_z}^{C_1}(\psi) = \begin{bmatrix} \cos\psi & \sin\psi & 0 \\ -\sin\psi & \cos\psi & 0 \\ 0 & 0 & 1 \end{bmatrix} \tag{4c} $$

${}^{C_2}{A_x}^{C_1}(\psi)$, ${}^{C_2}{A_y}^{C_1}(\psi)$, and ${}^{C_2}{A_z}^{C_1}(\psi)$, represent a rotation of $\psi$ about $x$, $y$, and $z$-axis, respectively, where $C_1$ and $C_2$ can be global or local CS in this work.

A convenient combination of the elemental CTMs is know as the Eulerian Transformation[1,2], and is defined as follows

$$ {}^{C_2}A^{C_1} = A_z(\gamma) \cdot A_x(\beta) \cdot A_z(\alpha) = \begin{bmatrix} A_{11} & A_{12} & A_{13} \\ A_{21} & A_{22} & A_{23} \\ A_{31} & A_{32} & A_{33} \end{bmatrix} \tag{5} $$

here, $(\alpha, \beta, \gamma)$ are "Eulerian Angles". The concept of Eulerian transformation is further illustrated in Fig. 2. As it can be seen in this figure, in order to transform $C_1\{\hat{x}_1, \hat{y}_1, \hat{z}_1\}$ to another arbitrarily

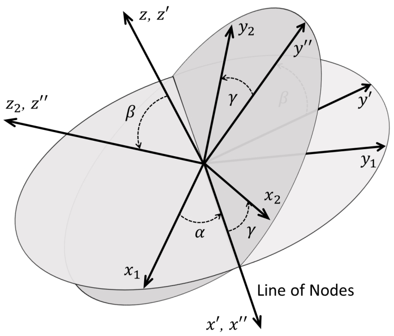


Figure 2: Eulerian Transformation. Line of Nodes represents the intersection of $x_1y_1$, and $x_2y_2$ planes [2].

oriented CS, $C_2\{\hat{x}_2, \hat{y}_2, \hat{z}_2\}$, one needs to first rotate the $C_1$ counter-clockwise (CCW) by an angle $\alpha$ about the $z_1$-axis, to make $x_1$ aligned with the "line of nodes" which is the intersection of $x_1y_1$, and $x_2y_2$ planes. The resulting CS is $C^{'}\{\hat{x}^{'}, \hat{y}^{'}, \hat{z}^{'}\}$. Next, $C^{'}$ should be rotated CCW by an angle $\beta$ about the line of nodes ($x^{'}$) to align $z^{'}$ with $z_2$, giving a new CS, $C^{''}\{\hat{x}^{''}, \hat{y}^{''}, \hat{z}^{''}\}$. Finally, $C^{''}$ is needed to be rotated CCW about $z^{''}$ ($Z_2$) by an angle $\gamma$ to align $C^{''}$ with $C_2$ [2].


1. Duan, D.-W. & Rahmat-Samii, Y. Novel coordinate system and rotation transformations for antenna applications. *Electromagnetics* **15**, 17–40 (1995).

2. Rahmat-Samii, Y. Useful coordinate transformations for antenna applications. *IEEE Trans. Antennas Propag.* **27**, 571–574 (1979).
